# Effects of Pollen Deprivation in Groups of Tellian (*Apis mellifera intermissa*) and Saharan (*Apis mellifera sahariensis*) Honey Bees under Controlled Conditions

**DOI:** 10.3390/insects13080727

**Published:** 2022-08-15

**Authors:** Hassiba Khedidji, Khaled Abderrahmani, Hakima Oulebsir-Mohandkaci, Kafia Ladjali-Mohammedi, Arezki Mohammedi

**Affiliations:** 1 Laboratory of Valorization and Conservation of Biological Resources, Faculty of Sciences, University M’hamed Bougara of Boumerdes, Boumerdes 35000, Algeria; 2 National Centre for Research and Development of Fisheries and Aquaculture (CNRDPA), Boulevard Colonel Amirouche Bouismail, Tipaza 42415, Algeria; 3 Laboratory of Cellular and Molecular Biology, University of Sciences and Technology Houari Boumediene (USTHB), El-Alia, Bab-Ezzouar, Algiers 16110, Algeria

**Keywords:** *Apis mellifera intermissa*, *Apis mellifera sahariensis*, pollen deficiency, ovary development, hypopharyngeal glands, hemolymph protein, lifespan

## Abstract

**Simple Summary:**

Pollen is a key element in bee nutrition, containing proteins, vitamins and lipids that support the vital functions of bees. Nutritional stress affects bees from the larval to the adult stage, both at the individual and colony levels. To face this type of stress, the bees developed behavioral and physiological mechanisms to fight against it. Such type of response can vary depending on the breed and age of the bees. In this study under controlled conditions, we investigated the impact of a protein-free diet on physiological functions, including survival, hypopharyngeal gland development, hemolymph proteins and ovarian development of two local bees, *Apis mellifera intermissa* and *Apis mellifera sahariensis*.

**Abstract:**

Worldwide, honey bees are increasingly faced with periods of pollen scarcity, which can lead to nutritional deficiencies, especially of proteins and amino acids. These are essential for the proper functioning of the single organism and the colony. To understand how bees react to protein deficiency, under controlled conditions, we studied the effect of pollen deficiency on the main physiological parameters in two subspecies endemic of Algeria, *Apis mellifera intermissa* and *Apis* *mellifera sahariensis*. Emerging workers of both subspecies were reared with two diets: one was pollen-fed, whereas the other pollen-deprived. Several physiological criteria were measured depending on the type of diet and subspecies: the survival of the bees, the amount of total protein in the hemolymph, hypopharyngeal glands development and the ovary development of workers. These last three parameters were assessed at three different ages (7, 14 and 21 days). At birth, *sahariensis* workers weighed more than *intermissa*. With the same protein diet, the average life expectancy of *sahariensis* was extended by 5.55 days compared to *intermissa*. Even if deprived of pollen, *sahariensis* lived longer than *intermissa* fed with pollen (*p* < 0.001). In the three age levels, the hypopharyngeal glands were more developed and less affected by pollen deficiency (*p* < 0.001) in *sahariensis* than in *intermissa* (*p* < 0.001). The total hemolymph protein was higher in *intermissa* than in *sahariensis* regardless of the diet, and was also higher in protein-fed than in deprived bees (*p* < 0.001). The ovaries developed more rapidly with a high proportion in *intermissa* than in *sahariensis* (*p* < 0.05) regardless of the diet, and was also higher in the bees fed with pollen than those deprived (*p* < 0.05). Pollen deficiency generates physiological alterations and modifications, the amplitude of which varied according to the subspecies of the bee studied.

## 1. Introduction

The natural habitat of the *Apis mellifera* bee extends from South Africa through the savannah, rainforest, desert and mild climate of the Mediterranean reaching the limit of its expansion in northern Europe and southern Scandinavia [1]. More than 25 sub-species of bees evolved within such a variety of habitats, climatic conditions and flora, with its own characteristic adaptations to each region [2,3]. Two endemic honey bee subspecies, *Apis mellifera intermissa* (Tellian bee) and *Apis mellifera sahariensis* (Saharan bee), are present in Algeria. *A. mellifera intermissa* inhabits the majority of Algerian apiaries, while *A. mellifera sahariensis* is confined to the southwestern region of Algeria [4]. *A. mellifera sahariensis* faces several threats that are of anthropic and natural origin, such as the drought that is increasingly frequent in the steppe and the Sahara. In addition, global warming could aggravate the already precarious situation of *A. mellifera sahariensis* [5].

The Intergovernmental Panel on Climate Change (IPCC) reports an approximate temperature increase in the range of 1.1–6.4 °C by the end of this century. This climate change can have a negative effect on the bee itself but also on its floral environment [6]. Excessively dry climate reduces pollen. which is the only source of proteins for bees [7]. Actually, the ratio and level of the major nutritional components of pollen, including proteins and their constituent amino acids, lipids (including flavonoids phytosterols), carbohydrates, and vitamins and secondary metabolites such as carotenoids, determine its nutritional value [8]. Pollen with good nutritional value provides high levels of hemolymph proteins [9] and ensures survival and good colony growth [10]. The need for protein is variable; it is linked to age, health and physiological status. The nurses require an adequate protein intake to be able to develop their hypopharyngeal glands (HGPs) and to synthesize royal jelly. Protein-rich pollen permits a good development of HGPs [11], improves the reproductive system, promotes ovarian development and increases drone fertility [12,13], boosts the immune defense [10] and increases the activity of digestive enzymes [14]. Consequently, worker bees that suffer from pollen deprivation have a shorter lifespan [15,16], start foraging earlier [17,18], have less developed hypopharyngeal glands [19], poor ovarian development [12], have lower flight efficiency [20], and have lower levels of vitellogenin and transferrin expression [21].

However, as *A. mellifera sahariensis* has been evolving for thousands of years in an arid and hostile environment, whose thermal amplitude varies from −4 °C in winter to more than 40 °C in summer, it is probable this honey bee subspecies have developed behavioral and physiological characteristics very different from *A. mellifera intermissa*, which evolves in a relatively more clement environment [22]. The objective of this work is to test this hypothesis by evaluating the physiological response of the two subspecies of bees when they are completely deprived of a source of pollen. The physiological parameters studied to evaluate this response are the level of protein in the hemolymph, the development of the hypopharyngeal glands and ovaries, and the lifespan of the bees. Apart from a few studies characterizing the *A. mellifera sahariensis* populations on morphometric and molecular aspects [2,23], very few scientific works have been devoted to the *A. mellifera sahariensis*. In this work, the physiology of the two main subspecies of *A. mellifera* endemic to North Africa is studied for the first time under controlled conditions.

## 2. Materials and Methods

The experiment was carried out between March and June 2020 in the apiary of the laboratory of The University of Boumerdes (UMBB).

### 2.1. Source of the Honeybees

The experiment was carried out on the two local subspecies of bees in Algeria. *A. mellifera intermissa* is typically used in the north of Algeria for beekeeping, and *A. mellifera sahariensis* by beekeepers in oases in southwest Algeria. The two subspecies can be differentiated by their differences in size and color. *A. mellifera sahariensis* is slightly smaller than *A. mellifera intermissa* and is yellowish-reddish, while *A. mellifera intermissa* is black in color [5]. The bees were taken from two experimental apiaries. The first apiary of *A. mellifera intermissa* colonies is located on the university campus, and the second apiary of *A. mellifera sahariensis* colonies is located in an oasis of Ain sefra. This region is a part of the natural habitat of *A. mellifera sahariensis*. The two apiaries were monitored in the same way and treated against varroa at the appropriate time. For the purposes of the experiment, we moved ten *A. mellifera sahariensis* colonies and placed them in the experimental apiary on campus.

Three healthy colonies from each subspecies were selected. From each colony, frames containing brood close to emergence were kept in wooden cages (50 × 25 × 14 cm) separately and incubated at 34.5 °C and 75% RH. The bees were collected from the frames during the 24 h following the incubation.

### 2.2. Average Weight of Emerging Bees

After the emergence of the workers, a batch made up of 100 to 120 individuals randomly taken of each subspecies were weighed. The average weight of each bee (expressed in mg) was calculated based on the total number of individuals weighed.

### 2.3. Experimental Design

One hundred emerging bees were transferred to the Plexiglas bioassay cages (cage type Pain) [24] (12 × 10 × 4 cm) equipped with transparent and removable sides, ventilation holes and two syringes of 2.5 mL, which were adapted as feeders to provide a water and sucrose solution (50% *w*/*w*). A piece of wax was hung in the middle of each cage near the sugar solution and water for the bees to cluster on. 

Hoarding bioassay cages were maintained at controlled conditions (34 ± 1 °C, 50–70% relative humidity) and kept in the dark to simulate conditions within the colony. Pollen balls were provided ad libitum for the control bees only; these balls were prepared by mixing pollen (fresh polyfloral harvested the previous year and stored in the freezer) with honey until a soft, cohesive paste was obtained. Water and sucrose were also provided ad libitum for all the cages. A pollen ball of 10 grams was offered to each cage; this quantity allowed the pollen to be constantly available to the bees. The quantity of pollen consumed by caged bees is determined by the difference between the weight of pollen supplied and left after consumption. The diet was provided periodically at 1 or 2 d intervals, and at these time intervals, dead bees were removed and counted. The dead bees and the total number of bees in each cage were considered in the estimation of consumption. The mortality of the bees was registered until the last bee.

Three cages each containing 100 newly emerged *A. mellifera intermissa* or *A. mellifera sahariensis* worker bees were registered for each of the following treatments.

Lot 1: *A. mellifera sahariensis* pollen-deprived (SBP−) 

Lot 2: *A. mellifera sahariensis* pollen-fed (control) (SBP+) 

Lot 3: *A. mellifera intermissa* pollen-deprived (TBP−) 

Lot 4: *A. mellifera intermissa* pollen-fed (control) (TBP+)

For statistical analyses of data, we considered the three repetitions of each treatment as a single lot (one lot consists of three cages that received the same treatment).

### 2.4. Hemolymph Extraction

After confinement and feeding on the different diets, the hemolymph of 7-, 14- and 21-day-old workers was collected from a small incision at the level of the 3rd dorsal tergite, using microcapillary tubes previously washed in a 0.1% (*w*:*v*) phenylthiourea solution in water. The bees were placed in separate 1.5 mL tubes and kept refrigerated until their hemolymph was collected 1–2 h later. The hemolymph of 10 workers fed in each cage was conserved in microcapillary tubes at −20 °C for the later determination of total protein. After the hemolymph was collected, each bee was stored at the same temperature (−20 °C) until the further measurement of the hypopharyngeal glands (HPG) acini and ovary development.

### 2.5. Total Protein

The quantification of the total protein was carried out using the Bradford method [25]. This is a colorimetric measurement method, based on a reaction between proteins and a dye, Coomassie Brilliant Blue G250 (BBC), which is a red-stranded reagent in the free state that turns blue when bound to proteins, the intensity of the colour being proportional to the concentration of the proteins. The Bradford reagent (BBC) was prepared from 50 mg of Coomassie Brilliant Blue, 25 mL of 95° ethanol, 50 mL of 85% orthophosphoric acid and 500 mL with distilled water. The calibration range was prepared from a stock solution of 10 mg/mL Bovine Serum Albumin (BSA) at 1 mg/mL and a stock solution of 0.1% BSA; then, a series of 6 tubes of increasing concentration were established and incubated for 10 min at room temperature in the dark. The optical density was measured using a spectrophotometer at a wavelength of 595 nm; a calibration curve was then drawn. Samples were thawed in fresh water before a series of tubes were prepared each containing 5 μL of hemolymph made up to 100 μL by adding 95 μL of distilled water. Subsequently, a volume of 4 mL of BBC was added to both sets of tubes, mixed and incubated for 10 min at 4 °C in the dark, before running through the spectrophotometer. The values obtained were plotted on the calibration curve already drawn to determine the protein concentration.

### 2.6. Extraction and Measurement of Hypopharyngeal Glands (HPG)

HPG were removed with forceps through an incision in the front part of the head and put on a microscope slide in a droplet of sodium chloride solution. The development of the HPG was assessed by calculating the area of the acini according to the protocol established by DeGrandi-Hoffman (2010) [26]. A photo of the glands was taken using a Leica DM 500 microscope with a Leica DC-300 camera and Axio-vision image manager software program. For calibration, a photograph of a 1 mm line was taken at the same magnification used for observing the HPG. The area of each acinus was calculated by multiplying the length by the width and considered as a parameter for development of the acini. Six acini per bee were selected and only acini with clear borders were measured. Acini areas were averaged to obtain an estimated HPG mean size for the cage. 

### 2.7. Ovary Assessments

The ovary development was assessed using a binocular microscope and the classification of the different developmental stages as proposed by Hess (1942) [27] and modified by Velthuis (1970) [28]. Five stages were differentiated: stage 1 is inactive thread-like ovarioles without vitellus; stage 2 is swollen ovarioles without vitellus; stage 3 is swollen ovarioles with visible vitellus but without distinct oocytes; in stage 4, the ovarioles contain distinct, but immature oocytes; and stage 5 is fully activated ovaries with distinct mature oocytes. The two first stages (sizes 1 and 2) correspond to undeveloped ovaries and the last three (sizes 3–5) to developed ovaries. 

### 2.8. Statistical Analysis

Statistical analysis of the data was conducted using the program Statistic, IBM SPSS Statistics 28.0. Results were expressed as means ± standard deviation. The weight of emerging bees was evaluated using a one-way ANOVA. The total protein of the hemolymph were analyzed by using a full-factorial ANOVA. The surface of the hypopharyngeal glands for the three age levels was reanalyzed using ANOVA three ways and Tukey’s HDS test for the post hoc multiple comparisons, to indicate the differences. The level of significance was determined at 5% (normal distribution of the data was verified using the Kolmogrov–Smirnov test before carrying out ANOVA). A chi^2^ test was performed to study the dependence of ovary development on subspecies and diet. 

The survival was analyzed using the Kaplan–Meier survival estimator, and Gehan–Wilcoxon test was conducted to determine the differences. Differences in pollen consumption were investigated using Student’s *t*-tests.

## 3. Results

### 3.1. Average Weight of Emerging Bees

At emergence, *sahariensis* weighed on average 133 ± 0.06 mg and *intermissa* 90 ± 0.05 mg (F_1,18_ = 252.57; *p* < 0.001).

### 3.2. Food Consumption

Pollen consumption was statistically the same for bees of both the subspecies (F_1,4_ = 0.732; *p* > 0.05): on average, pollen consumption was 2.62 ± 0.33 mg/day for *A. mellifera intermissa* and 2.13 ± 0.61 mg/day for *A. mellifera sahariensis*.

### 3.3. Total Hemolymph Protein

**Figure 1:**
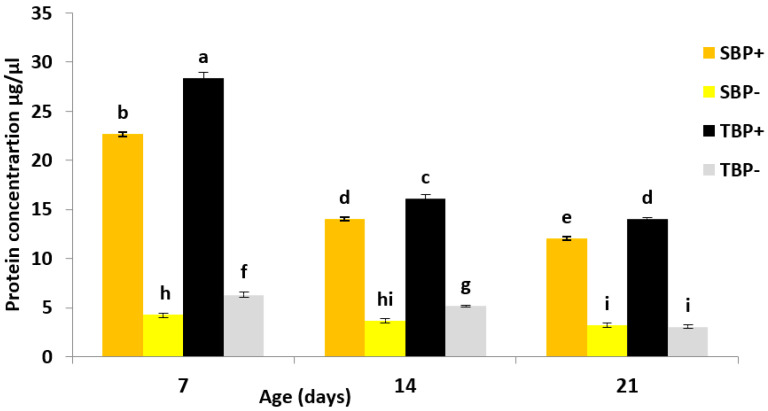
Mean ± SE protein concentration in hemolymph of both the subspecies of bees at 7, 14 and 21 days. SBP+: *A. mellifera sahariensis* with pollen, SBP−: *A. mellifera sahariensis* pollen-deprived, TBP+: *A. mellifera intermissa* with pollen, TBP−: *A. mellifera intermissa* pollen-deprived. The error bars represent the standard deviation from the respective means. Bars with the same letter are not significantly different (*p* < 0.05).

The amount of protein in the hemolymph (HP) was influenced by the diet (F_1,96_ = 23,005.41; *p* < 0.001), the subspecies (F_1,96_ = 597.62; *p* < 0.001) and age (F_2,96_ = 2436.39; *p* < 0.001).The statistical interactions diet × subspecies (F_1,96_ = 137.57; *p* < 0.001), subspecies × age (F_2,96_ = 97.16; *p* < 0.001), diet × age (F_2,96_ = 1378.77; *p* < 0.001) and subspecies × diet × age (F_2,96_ = 24.40; *p* < 0.001) were also significant (Figure 1).

At 7 days, TBP+ had a significantly higher HP content than TBP− (Tukey’s HDS test; *p* < 0.001) and SBP+ (Tukey’s HDS test; *p* < 0.001). SBP+ had significantly more HP than SBP− (Tukey’s HDS test; *p* < 0.001). In the situation of pollen deficiency, the HP content was significantly higher in TBP− than in SBP− (Tukey’s HDS test; *p* < 0.001).

At 14 days, the amount of HP decreased significantly within all lots and followed the same trend among the different batches. TBP+ had significantly more HP than TBP− (Tukey’s HDS test; *p* < 0.001) and SBP+ (Tukey’s HDS test; *p* < 0.001). SBP+ had significantly more HP than SBP− (Tukey’s HDS test; *p* < 0.001). In a situation of pollen deficiency, the amount of HP was significantly higher in TBP− than in SBP− (Tukey’s HDS test; *p* < 0.001).

At 21 days, a slight decrease was observed in the amount of HP compared to 14-day-old bees, with the same trend between the four groups. TBP+ had a significantly higher quantity of HP than TBP− (Tukey’s HDS test; *p* < 0.001) and SBP+ (Tukey’s HDS test; *p* < 0.001). In the batches of bees deprived of pollen, more HP in SBP− than in TBP− was observed; however, the difference was not significant (Tukey’s HDS test; *p* > 0.05).

### 3.4. Hypopharyngeal Glands’ Development

**Figure 2:**
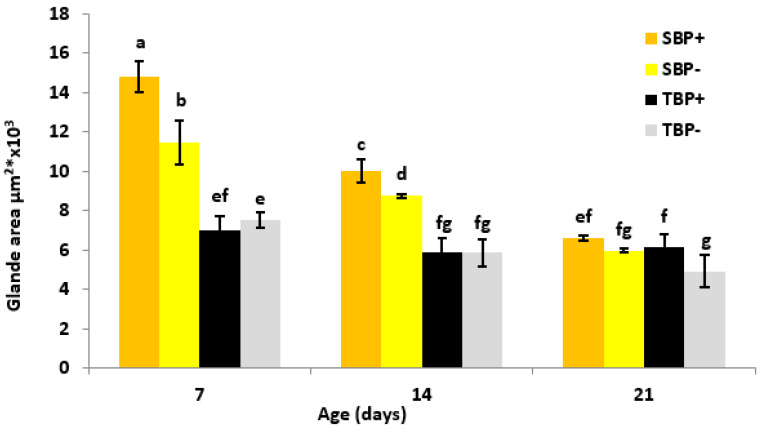
Mean ± SE acini surface area of the hypopharyngeal glands of both the subspecies of bees, at 7, 14 and 21 days. SBP+: *A. mellifera sahariensis* pollen-fed, SBP−: *A. mellifera sahariensis* pollen-deprived, TBP+: *A. mellifera intermissa* pollen-fed, TBP−: *A. mellifera intermissa* pollen-deprived. The error bars represent the standard deviation for the mean area of the hypopharyngeal glands. Bars with the same letter are not significantly different (*p* < 0.05).

The development of the HPGs was influenced by the diet (F_1,348_ = 43.69; *p* < 0.001), the subspecies (F_1,348_ = 508.45; *p* < 0.001), the age (F_2,348_ = 278.43; *p* < 0.001), the diet × subspecies interactions (F_1,348_ = 29.99; *p* < 0.001), the subspecies × age (F_2,348_ = 97.05; *p* < 0.001) and the subspecies × diet × age interactions (F_2,348_ = 18.66; *p* < 0.001). However, the effect of the diet × age interactions was not significant (F_3,348_ = 2.03; *p* < 0.132) (Figure 2).

At 7 days of age, the HPG of SBP+ were significantly more developed than those of SBP− (Tukey’s HDS test; *p* < 0.001) and TBP+ (Tukey’s HDS test; *p* < 0.001). The HPGs of SBP− were significantly more developed than those of TBP− (Tukey’s HDS test; *p* < 0.001). However, in *intermissa*, there was no difference between TBP+ and TBP− (Tukey’s HDS test; *p* > 0.05).

At 14 days, the HPG of SBP+ were significantly more developed than those of TBP+ (Tukey’s HDS test; *p* < 0.001) and SBP− (Tukey’s HDS test; *p* < 0.033), whereas the HPGs of SBP− were significantly more developed than those of TBP− (Tukey’s HDS test; *p* < 0.001). In *intermissa*, there was no significant difference between TBP+ and TBP− (Tukey’s HDS test; *p* > 0.05).

At 21 days, HPG atrophy was observed in *sahariensis* (SBP+ and SBP−). At this age, there were no significant differences between the groups except in *intermissa* where the HPG of TBP+ were significantly more developed than those of TBP− (Tukey’s HDS test; *p* = 0.045).

### 3.5. Ovary Development

**Figure 3:**
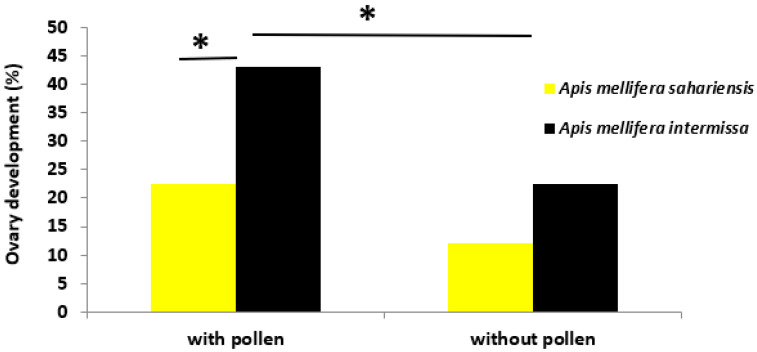
Ovary development rate in both subspecies of bee (*Apis mellifera intermissa* and *Apis mellifera sahariensis)*, pollen-fed (control) and pollen-deprived. The asterisk (*) indicates the significance of the chi-squared (χ^2^) test at a significance level of *p*-value < 0.05.

Ovary development was greater in *intermissa* than in *sahariensis*. It also depended on the diet of the bees; whether it was rich or deficient in pollen. The result of the chi^2^ test therefore indicated a dependence of ovary development on diet and subspecies (χ^2^_(3)_ = 14.05; *p* < 0.05) (Figure 3).

A protein diet had a significant effect on the development of the ovaries in *intermissa*. In TBP+, there were significantly more developed ovaries than in TBP− (χ^2^_(1)_ = 4.804; *p* < 0.05). The comparison between subspecies showed that TBP+ had significantly more developed ovaries than SBP+ (χ^2^_(1)_ = 4.804; *p* < 0.028).

On the other hand, the diet did not seem to have an influence on the ovary development of *sahariensis* since there was no significant difference in the ovary development of SBP+ and SBP− (χ^2^_(1)_ = 2.0235; *p* > 0.05). When they were both deprived of pollen, there was no significant difference in the ovary development of SBP− and TBP− (χ^2^_(1)_ = 2.025; *p* > 0.05). 

### 3.6. Bee Survival

**Figure 4:**
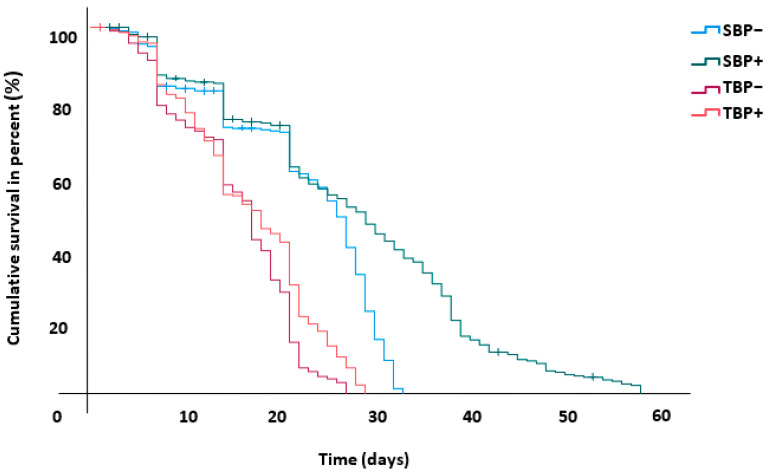
Cumulative survival in percent of worker bees. SBP+: *A. mellifera sahariensis* pollen-fed, SBP−: *A. mellifera sahariensis* pollen-deprived, TBP+: *A. mellifera intermissa* pollen-fed, TBP−: *A. mellifera intermissa* pollen-deprived.

The survival analysis was conducted over a period of 58 days, the period that corresponded to the date from the start of the trial until the death of the last bee. The Kaplan–Meier estimation results revealed that the survival of *sahariensis* was superior to *intermissa* even with a pollen-free diet (Figure 4, Table 1). All pairwise comparisons between groups were significantly different. 

SBP+/TBP+ (Wilcoxon test, χ^2^_(1)_ = 105.65; *p* < 0.001)

SBP−/TBP− (Wilcoxon test, χ^2^_(1)_ = 119.61; *p* < 0.001)

SBP+/SBP− (Wilcoxon test, χ^2^_(1)_ = 24.286; *p* < 0.001)

TBP+/TBP− (Wilcoxon test, χ^2^_(1)_ = 6.803; *p* < 0.009)

## 4. Discussion

This study showed that pollen consumption has an influence on the amount of protein in the hemolymph of caged bees. However, it also depends on the subspecies since, when the same amount of pollen is consumed, *intermissa* has more hemolymphatic proteins than *sahariensis*. These results suggest that *intermissa* optimizes its protein diet much better than *sahariensis*. A study has shown that, for the same pollen source, the Egyptian subspecies *Apis mellifera lamarckii* has a higher protein content in its hemolymph than the two subspecies *Apis mellifera carnica* and *Apis mellifera ligustica* [29]. In that study, the authors explain this difference by the greater enzymatic secretion in the intestine. In a subsequent study carried out on pre-pupae of *Apis mellifera* [30], the researchers demonstrated the role of the enzyme acid phosphatase, which would be at the origin of the accumulation of proteins in the hemolymph and in the mobilization of proteins stored in the trophocytes to be used during metamorphosis. Another study [31] has also shown the superiority of Africanized bees in the conversion of pollen proteins into hemolymph proteins compared to European bees. It is therefore possible that *intermissa* secretes more acid phosphatase enzyme in its intestine than *sahariensis*. As the two subspecies have evolved under different floral environment, they are maybe more adapted to digest specific pollen diets, and so the diet used in our study may be more adapted to *intermissa*.

This enzyme could also play a role in the mobilization of body proteins to store them in the hemolymph since even in a situation of pollen deficiency *intermissa* has more hemolymphatic proteins than *sahariensis*.

Once stored in the bee’s hemolymph, nutrients (proteins, carbohydrates, lipids and vitamins) as well as hormones are distributed according to various physiological needs [32,33]. These proteins play an essential role in the functioning of the hypopharyngeal glands [12,19,26,34]. This study shows that, even if deprived of pollen, *sahariensis* has more developed hypopharyngeal glands than *intermissa* fed with pollen. This trend is observed at 7 and 14 days. This is an age class that corresponds to that of nurses [35]. Field experiments have shown that *sahariensis* produces more royal jelly than *intermissa* (unpublished data). These results could support the hypothesis that *sahariensis* gives priority to the development of the hypoharyngeal glands in order to nourish the brood in an optimal way. This priority would even be to the detriment of the body reserves of the pollen-deficient nurse bee.

According to [13], bees fed a high protein diet have more developed ovaries. This is in agreement with our results, which clearly show that pollen fed bees have the highest proportion of developed ovaries than those fed only with sugar syrup.

However, ovarian development does not depend solely on a diet rich in protein since [36] also demonstrates the role of carbohydrates in ovarian development; the author indicated that a diet with a protein/carbohydrate ratio of (1:3) results in better ovarian development. Even without a protein diet, a greater proportion of developed ovaries is recorded in *intermissa*. It therefore seems that ovarian development does not only depend on diet but also depends on subspecies. When the queen and brood are experimentally removed, *intermissa* develops its ovaries more rapidly (5.6 days) than the African subspecies *scutellata* (9.5 days) or European *carnica* (30 days) [37]. Likewise, *intermissa* lays more eggs than *scutellata* or *carnica*. The racial aspect is therefore important in the development of the workers ovaries [38].

In our experiment under controlled conditions, the bees of both subspecies consumed the same amount of pollen. The importance of this protein intake in the development of the *sahariensis* hypopharyngeal glands and the *intermissa* ovary development is shown. This protein supply is also important in the lifespan of bees [7,39]. *sahariensis* is much more affected by pollen deficiency than *intermissa*. Indeed, when the bees are pollen deprived, the life expectancy of *intermissa* and *sahariensis* is shortened by just over 8.5% and 18.83%, respectively. On the other hand, when the bees are pollen fed, the average life expectancy of *sahariensis* is extended by 5.55 days compared to *intermissa*. It is important to note that even when deprived of pollen, *sahariensis* lives longer than pollen-fed *intermissa*. This difference in life expectancy between the two subspecies could have its origin during the larval development of the bee. Indeed, we found that at emergence, *sahariensis* weighed more than *intermissa*. This weight difference could be due to a good diet during the larval phase. This hypothesis is confirmed by the propensity of *sahariensis* to promote the development of its hypopharyngeal glands to the detriment of its ovaries.

Numerous studies have also shown that the phospholipoglycoprotein vitellogenin, known as female-specific egg yolk proteins, accumulates in body reserves and in the hypopharyngeal glands and has a positive effect on the longevity of workers [40,41,42,43,44]. In a recent study carried out on honey bee workers [45], it is demonstrated that telomerase activity and the production of the hormone vitellogenin are positively correlated with body mass and fat cell size. Telomeres, which are involved in the process of survival and aging in eukaryotes [46], bind the ends of chromosomes. They are shortened under the effect of oxidative stress and during cell division [47]. It is possible that, in the case of *sahariensis*, the oxidative stress is minimal enough not to negatively affect its life expectancy.

These results obtained under controlled conditions made it possible for the first time to highlight certain physiological characteristics of the two subspecies of bees endemic to North Africa. 

The two subspecies evolve in two different environments. *Intermissa* lives in regions characterized by a Mediterranean climate where honey flora is abundant almost all year round. The opulence that benefits *intermissa* means that this subspecies uses nutrients to promote excessive reproduction [38], while the desert environment of *sahariensis* is very hostile. 

Moreover, it could be said that, in this environment, *sahariensis* do not have interest to engage in ovary development for workers as it can be a loss of energy for the whole colony.

The *sahariensis* subspecies seems to be endowed with physiological qualities allowing it to face a hostile environment. Long periods of scarcity are recurrent in the Sahara and *sahariensis* has learned to live in this environment. Optimal feeding of the larvae as well as longevity of the workers could be a response to this hostility of the nature.

## Figures and Tables

**Table 1:** Kaplan–Meier estimate of survival time of *Apis mellifera sahariensis* and *Apis mellifera intermissa* according to diet (pollen-fed and pollen-deprived), with confidence interval (95% CI), results expressed as (mean ± standard error), and letters indicate significant difference test (Gehan–Wilcoxon test).

	Mean Survival ± Standard Error	
Diet	*Apis mellifera sahariensis*	*Apis mellifera intermissa*
With pollen	27.50 ± 0.78 a	16.76 ± 0.41 c
Without pollen	22.31 ± 0.50 b	15.33 ± 0.36 d

## Data Availability

Data are available upon request from the corresponding author.
